# COVID-19: Needs-led implementation and the immediate potential of remote monitoring

**DOI:** 10.3399/bjgpopen20X101093

**Published:** 2020-05-20

**Authors:** Nikki Newhouse, Andrew Farmer, Maxine E Whelan

**Affiliations:** 1 Postdoctoral Research Fellow, Nuffield Department of Primary Care Health Sciences, University of Oxford, Oxford, UK; 2 Professor of General Practice, Nuffield Department of Primary Care Health Sciences, University of Oxford, Oxford, UK; 3 Postdoctoral Researcher, Nuffield Department of Primary Care Health Sciences, University of Oxford, Oxford, UK

**Keywords:** Continuity of care, Respiratory illness, Information technology, Primary health care, General practice

An ongoing outbreak of coronavirus (COVID-19) is causing public health concern on a global scale.^1^ With substantial person-to-person transmission, special attention is needed to reduce transmission and to protect susceptible populations, including older people who live with weakened immune systems.^2^

As a result of this viral outbreak, the world as we know it is changing and barriers to delivering health care remotely are disappearing rapidly. Projects long in the planning are leapfrogging developmental stages straight into implementation, driven by the urgent need to share healthcare information to mitigate challenges raised by social distancing. For those of us interested in exploring remote ways to support patient care in the primary care setting, the COVID-19 global pandemic presents a unique natural experiment that can inform future best practice for digital health care.

Swift additions to the technological healthcare ‘toolkit’ include video-based consultation,^3^ provision of tablet computers to facilitate ‘virtual visits’,^4^ and telephone-first assessment. Patients and clinicians alike are embracing digital health. A survey reported that 38% of responders increased their use of NHS technology since the start of the outbreak.^5^ People with suspected COVID-19 symptoms have been receiving regular check-ins from an NHS messaging service. The NHS app has been downloaded 434 000 times since 24 February 2020,^6^ offering a gateway to NHS services; patients can use the app to book appointments and view medical records.

Community-based initiatives and digital technologies provide solutions to challenges arising from the COVID-19 pandemic. This current activity also provides an opportunity to reflect on and rethink our approach to how we conduct consultations with patients. While the default face-to-face format has been subject to debate, the sudden implementation of technological alternatives will likely impact discussion around its future role. With the majority of people being asked to stay away from GP practices, we should now be discussing the value of returning to this model of care.

However, this is not a call for ‘digital exceptionalism’; significant barriers to uptake and implementation of digital solutions remain, not least around appropriate evaluation, electronic surveillance culture, equitable access, mitigation of health inequalities, cost, and the vital importance of face-to-face contact. Indeed, despite the immediate potential of digital medicines to support health care, it is imperative that we avoid falling into the reactive trap of believing that digital ‘solutionism’^7^ is the only way forward. Digital ‘fixes’ cannot simply replace meaningful political and social change, and must not become the default option as we emerge from the current crisis. Digital health technologies are not inherently positive and progressive, and the UK’s track record in the successful delivery of large-scale digital projects has been somewhat inconsistent.

Nonetheless, telemedicine is not a novel concept and using technology to support care delivery is relatively commonplace, particularly in rural and remote settings. As early as 1998, nurses and GPs in a rural village in Scotland, UK were able to support physical ailments, mental health problems, and discussions with the doctor from afar.^8^ Use of technology to reach isolated communities has been developed to overcome geographical problems, and can save time and travelling for patients.^9^

Key evidence for the value in shifting health work online also comes from the field of personal health informatics, which has long pressed for a shift towards the remote monitoring of vital signs. Personal health informatics involves collecting personally relevant information with the intention of increasing knowledge, supporting more traditional forms of care delivery. The value of this is immediately apparent in the current climate. Alongside telephone or video consultation, digital self-monitoring tools provide information previously available only with face-to-face evaluation. Remote assessment can therefore keep at-risk patients away from healthcare settings where there is risk of potential infection. In addition, such tools can deliver crucial information to guide remote diagnostics and monitoring. In particular, the application of remote monitoring could play a central role in caring for vulnerable patient populations at risk of rapid deterioration, for example those living with chronic obstructive pulmonary disease (COPD).

Drawing on our work involving patients with COPD, we have explored the role of a tablet computer in supporting self-management. Using the EDGE system,^10^ patients can complete symptom diaries, measure their oxygen saturation levels and pulse daily, and wear a physical activity monitor, with all output monitored remotely by a clinical team, who are alerted to sustained, abnormal values. Personalised videos encouraging good inhaler technique and self-care facilitate engagement with the device. Patient feedback indicates that using the system is acceptable to patients and healthcare teams.

The urgent implementation and embedding of technological alternatives to face-to-face care has been ‘needs-led’, and key data needs to be captured with regard to best use of remote monitoring as we prepare for a ‘new normal’ in primary care (Box 1).

Box 1 **Key considerations to inform remote monitoring in the ‘new normal’ of primary care.**

**Initiation:** The variation in remote monitoring practices may not feel immediately ‘successful’ but people are driving this forward.

· What can we get into practice now?

· What aspects of remote monitoring need to be improved and for whom in particular?

**Differentiation:** People are exploring new ways of working and there is likely to be wide variation in how remote monitoring is being conducted, varying by practice and by patient group.

· What can we learn from this variation?

· How can we harness best practice?

**Relational integration:** The immediate need to implement new ways of working will challenge individual’s views of the value and praxis of remote monitoring. Opinion around how remote monitoring can ‘fit’ within primary care may change and it may cause some disruption.

· What opinions change?

· Which concerns persist about how and why we might use technology in this way?

**Reconfiguration:** The paradigm of primary care consultations is rapidly evolving and there will be learning opportunities to inform the future.

· What does an optimal future look like?

· What should we retain?

Harnessing the willingness to implement remote monitoring in primary care in this time of public health crisis is an opportunity we cannot let pass us by. We are living in a time of unparalleled challenge, but one which also offers a unique opportunity for exploration. Driven by the urgent need to monitor vital signs and other clinical measurements alongside a telephone or video consultation, we need to begin experimenting with how to best deliver this, and it is apt to start now during the COVID-19 pandemic while there is momentum to do so.
